# Calcium dysregulation in atrial fibrillation: the role of CaMKII

**DOI:** 10.3389/fphar.2014.00030

**Published:** 2014-03-04

**Authors:** Jordi Heijman, Niels Voigt, Xander H. T. Wehrens, Dobromir Dobrev

**Affiliations:** ^1^Institute of Pharmacology, Faculty of Medicine, University Duisburg-EssenEssen, Germany; ^2^Cardiovascular Research Institute, Departments of Molecular Physiology and Biophysics, and Medicine-Cardiology, Baylor College of MedicineHouston, TX, USA

**Keywords:** atrial fibrillation, calcium, CaMKII, ectopic activity, reentry

## Abstract

Atrial fibrillation (AF) is the most frequently encountered clinical arrhythmia and is associated with increased morbidity and mortality. Ectopic activity and reentry are considered major arrhythmogenic mechanisms contributing to the initiation and maintenance of AF. In addition, AF is self-reinforcing through progressive electrical and structural remodeling which stabilize the arrhythmia and make it more difficult to treat. Recent research has suggested an important role for Ca^2+^-dysregulation in AF. Ca^2+^-handling abnormalities may promote ectopic activity, conduction abnormalities facilitating reentry, and AF-related remodeling. In this review article, we summarize the Ca^2+^-handling derangements occurring in AF and discuss their impact on fundamental arrhythmogenic mechanisms. We focus in particular on the role of the multifunctional Ca^2+^/calmodulin-dependent protein kinase type-II (CaMKII), which acts as a major link between Ca^2+^-dysregulation and arrhythmogenesis. CaMKII expression and activity are increased in AF and promote arrhythmogenesis through phosphorylation of various targets involved in cardiac electrophysiology and excitation-contraction coupling. We discuss the implications for potential novel therapeutic strategies for AF based on CaMKII and Ca^2+^-handling abnormalities.

## Introduction

Atrial fibrillation (AF) is the most prevalent heart-rhythm disorder, estimated to affect more than 33 million people worldwide (Chugh et al., 2013). AF is associated with increased morbidity and mortality, notably as a risk factor for stroke and worsening of heart failure (Camm et al., 2012; Chugh et al., 2013). Current pharmacological treatments for rhythm-control of AF mainly include class-I and class-III antiarrhythmic drugs, which have modest efficacy, providing sinus-rhythm maintenance in only 30–70% of patients after >1 year of follow-up (Camm, 2012). In addition, these drugs are associated with substantial adverse side-effects including ventricular proarrhythmia and extra-cardiac toxicity (Zimetbaum, 2012; Heijman et al., 2013a). The AF incidence is expected to increase due to aging of the population, making the development of improved antiarrhythmic treatments of critical importance. A better understanding of AF pathophysiology is expected to foster this development (Dobrev et al., 2012). Accumulating evidence has highlighted a central role for abnormal Ca^2+^-handling in AF-pathophysiology (Dobrev and Nattel, 2008; Heijman et al., 2012; Nattel and Dobrev, 2012). Here, we review recent studies detailing the proarrhythmic role of AF-related Ca^2+^-handling abnormalities, with particular focus on the contributions of the Ca^2+^/calmodulin-dependent protein kinase type-II (CaMKII).

## Atrial cellular electrophysiology and arrhythmogenic mechanisms

### Normal atrial cellular electrophysiology and Ca^2+^-handling

The atrial action potential (AP) is determined by depolarizing and repolarizing ionic currents (Dobrev and Ravens, 2003). Depolarizing currents include the cardiac voltage-gated Na^+^-current (I_Na_) and its persistent (“late”) component (I_Na,late_), the L-type Ca^2+^-current (I_Ca,L_) and the Na^+^/Ca^2+^-exchanger type-1 (NCX1) current (I_NCX_), which, in its forward mode, extrudes one Ca^2+^-ion in exchange for 3 Na^+^-ions, resulting in a net depolarizing inward current. Repolarizing currents include the transient-outward K^+^-current (I_to_), delayed-rectifier K^+^-currents with slow, rapid or ultra-rapid kinetics (I_Ks_, I_Kr_, and I_Kur_, respectively), as well as the Na^+^/K^+^-ATPase current (I_NaK_). In addition, AP duration (APD) and resting membrane potential are influenced by basal and acetylcholine-activated inward-rectifier K^+^-currents (I_K1_ and I_K,ACh_). The I_Kur_ and I_K,ACh_ currents are predominantly expressed in the atria, thereby providing potential atrial-specific therapeutic targets.

Ca^2+^ entry through the L-type Ca^2+^-channel activates Ca^2+^-induced Ca^2+^-release from the sarcoplasmic reticulum (SR) through type-2 ryanodine receptor channels (RyR2), producing the systolic Ca^2+^-transient responsible for initiating contraction of atrial cardiomyocytes (Bers, 2002). In addition, inositol 1,4,5-triphosphate (IP_3_)-receptor-mediated Ca^2+^-release may contribute to Ca^2+^-induced Ca^2+^-release by activating neighboring RyR2, although direct IP_3_-receptor-mediated activation of NCX1 has also been described recently (Dobrev and Nattel, 2008; Roderick and Knollmann, 2013).

Structural differences between atrial and ventricular cardiomyocytes may further contribute to a unique atrial Ca^2+^-handling profile. Isolated atrial cardiomyocytes generally have a less well-developed T-tubular network than ventricular cardiomyocytes. However, cardiomyocytes of certain species including humans, sheep, goats, cows, and horses do have more T-tubules than those from rodents (Dibb et al., 2009; Lenaerts et al., 2009; Richards et al., 2011). At least in sheep, this T-tubular system contributes to a more uniform, ventricular-like, Ca^2+^-induced Ca^2+^-release (Dibb et al., 2009). Although a small T-tubular system is present in human atrial myocytes, it shows some variability depending on region and cardiomyocyte size (Trafford et al., 2013). Moreover, this T-tubular system can be remodeled by cardiac disease including AF (Lenaerts et al., 2009). In atrial cardiomyocytes with a less well-developed T-tubular structure, Ca^2+^-induced Ca^2+^-release starts at the plasma membrane and propagates slowly toward the cell-center (Dobrev et al., 2009; Bootman et al., 2011). Relaxation occurs when Ca^2+^ is extruded from the cell via NCX1 and the plasmalemmal Ca^2+^-ATPase (PMCA), or is taken back up into the SR by the type-2a SR Ca^2+^-ATPase (SERCA2a). The affinity of SERCA2a for intracellular Ca^2+^ is largely determined by the inhibitory proteins phospholamban (PLB) and sarcolipin. The expression of sarcolipin is atrial-specific, whereas PLB is more strongly expressed in the ventricles than in the atria (Dobrev et al., 2009).

### Arrhythmogenic mechanisms in AF

AF can occur as a result of abnormalities in electrical impulse formation or impulse conduction (Nattel et al., 2008; Wakili et al., 2011; Heijman et al., 2014). Electrical impulse generation outside of the sinoatrial node, termed ectopic activity, can sustain AF as a driver, and can trigger reentry in a vulnerable substrate characterized by a slow and inhomogeneous conduction and short effective refractory periods. This vulnerable substrate can arise from genetic conditions, normal aging, or co-morbidities such as heart failure or hypertension (Wakili et al., 2011). Reentry can occur around anatomical obstacles or can be functional (i.e., occurring in the absence of anatomical obstacles). Reentry is considered the predominant mechanism for AF maintenance. When AF is maintained, atrial tachycardia-related remodeling produces electrical and structural alterations that further promote AF maintenance and stabilization, contributing to the progression toward longer-lasting AF episodes that are more difficult to treat.

At the cellular level, the effective refractory period is determined by APD and post-repolarization refractoriness. Conduction velocity is influenced by the depolarizing force through I_Na_, and the electrical conduction between atrial cardiomyocytes is controlled by gap-junction channels as well as the structure of the atrial myocardium, notably the amount and composition of the extracellular matrix, particularly fibrosis. The cellular mechanisms of ectopic activity mainly involve early and delayed afterdepolarizations (EADs and DADs, respectively). EADs are caused primarily by recovery from inactivation of I_Ca,L_ during excessive APD-prolongation, for example due to loss of repolarizing K^+^-currents. DADs are likely the most common mechanism underlying ectopic (triggered) activity and result from intracellular Ca^2+^-handling abnormalities. Spontaneous diastolic SR Ca^2+^-release events resulting from SR Ca^2+^-overload or intrinsic RyR2-dysfunction can activate NCX1, resulting in a transient-inward current that depolarizes the membrane potential as Ca^2+^ is extruded from the atrial cardiomyocyte (Dobrev and Wehrens, 2010). When the threshold for excitation is reached in a sufficient number of cardiomyocytes, an ectopic impulse is generated (Wakili et al., 2011).

## Structure, activation and targets of CaMKII

CaMKII is a multifunctional serine/threonine protein kinase that is abundantly expressed in various tissues including the heart (Swaminathan et al., 2012). There are four CaMKII isoforms, with CaMKIIδ being the most abundant in heart. CaMKIIδ has a hypervariable region, giving rise to multiple splice variants, including a splice variant with a nuclear localization signal (NLS; CaMKIIδ_B_) and one without such NLS sequence (CaMKIIδ_C_). The latter was traditionally considered cytosolic (Swaminathan et al., 2012), although this localization is not absolute (Mishra et al., 2011). CaMKII is a holoenzyme consisting of two stacked hexameric rings of subunits. Each subunit has a catalytic domain that, under resting conditions, is inhibited by regulatory domains of neighboring subunits. When intracellular Ca^2+^-levels periodically rise during the cellular Ca^2+^-transient, Ca^2+^ binds to calmodulin and activates CaMKII by binding to the regulatory domain (Swaminathan et al., 2012). CaMKII subunits can auto-phosphorylate Thr287 on neighboring subunits, thereby hindering the re-association of the catalytic and regulatory domains, producing sustained Ca^2+^-independent activation. This mechanism makes CaMKII activation strongly heart rate-dependent, with accumulating activity at faster rates. Furthermore, CaMKII can show Ca^2+^-independent activation following oxidation of Met281/282 by reactive oxygen species (Erickson et al., 2008), via O-linked glycosylation of Ser280 by O-linked N-acetylglucosamine (Erickson et al., 2013), and via NO-dependent nitrosylation of Cys116, Cys273, or Cys290, the exact residue being at present unknown (Gutierrez et al., 2013). In contrast, phosphorylation of Thr306/307 promotes CaMKII inactivation by reducing the binding of Ca^2+^/calmodulin complexes (Colbran, 1993).

**Figure 1:**
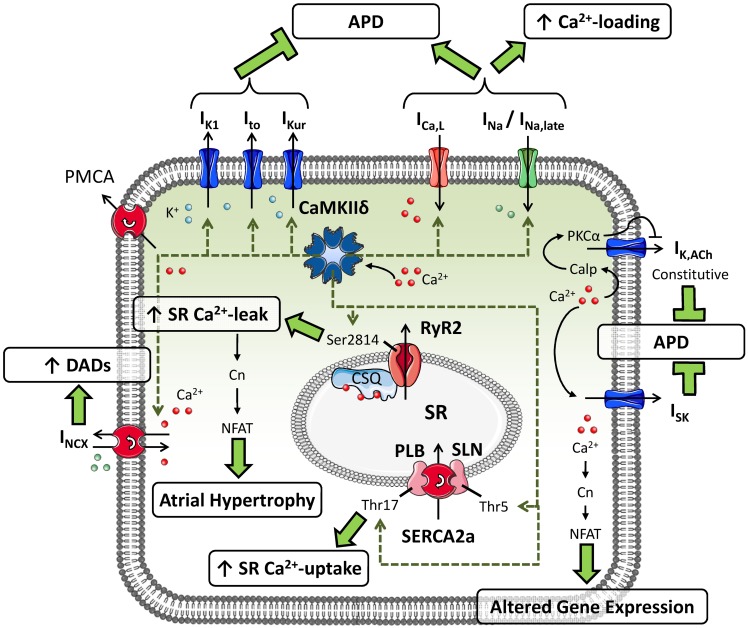
**Putative substrates for CaMKII-dependent phosphorylation in atrial cardiomyocytes and their consequences for atrial cellular electrophysiology and Ca^2+^-handling.** CaMKII can phosphorylate the transient-outward K^+^-current (I_to_), inward-rectifier K^+^-current (I_K1_) and ultra-rapid delayed-rectifier K^+^-current (I_Kur_), augmenting their functions and shortening action potential duration (APD). Phosphorylation of L-type Ca^2+^-current (I_Ca,L_) and Na^+^-current (I_Na_; resulting in an increased late component: I_Na,late_) by CaMKII increases intracellular Ca^2+^ levels and prolongs APD. CaMKII-dependent phosphorylation of phospholamban (PLB) and sarcolipin (SLN) increases sarcoplasmic reticulum (SR) Ca^2+^-uptake, whereas phosphorylation of type-2 ryanodine-receptor channels (RyR2) promotes diastolic SR Ca^2+^-leak. CaMKII-dependent increases in expression of Na^+^/Ca^2+^-exchanger type-1 (NCX1) augment NCX-current (I_NCX_), promoting the occurrence of delayed afterdepolarizations (DADs). In addition, Ca^2+^-handling abnormalities can activate small-conductance Ca^2+^-activated K^+^-currents (I_SK_) and agonist-independent “constitutive” I_K,ACh_, shortening APD, and promote altered gene expression via the Ca^2+^-dependent phosphatase calcineurin (Cn).

CaMKII can phosphorylate multiple substrates in atrial cardiomyocytes (Figure 1). CaMKII-dependent phosphorylation of L-type Ca^2+^-channels produces high-activity mode-2 gating resulting in increased open probability of I_Ca,L_, thereby augmenting the amount of Ca^2+^ entering the atrial cardiomyocyte. CaMKII also contributes to the increase in I_Ca,L_ following repeated depolarizing pulses (termed Ca^2+^-dependent I_Ca,L_-facilitation) (Swaminathan et al., 2012). CaMKII-dependent phosphorylation of Nav1.5 slows I_Na_ inactivation and augments the non-inactivating, “late” component of I_Na_ (Wagner et al., 2011). The Kv4.3 pore-forming subunit of I_to_ is also regulated by CaMKII-dependent phosphorylation through the accessory protein SAP97, resulting in increased I_to_ that would tend to shorten APD (El-Haou et al., 2009; Wagner et al., 2009). Based on experiments involving CaMKII inhibition with an inhibitory peptide or the experimental drug KN-93, CaMKII also appears to acutely augment I_K1_ (Wagner et al., 2009) and I_Kur_ (Tessier et al., 1999), thereby offsetting the APD-prolonging effects of CaMKII-dependent I_Ca,L_ and I_Na_ phosphorylation. In addition, both PLB and sarcolipin can undergo CaMKII-dependent phosphorylation, causing disinhibition of SERCA2a and increasing SR Ca^2+^-reuptake (Dobrev and Wehrens, 2010). Finally, CaMKII-dependent hyperphosphorylation of Ser2814 on RyR2 increases channel open probability, augmenting SR Ca^2+^-release. Taken together, CaMKII plays a nodal role in the modulation of atrial cellular Ca^2+^-handling.

## Ca^2+^/CaMKII dysregulation in AF

### Mechanisms promoting CaMKII dysregulation in AF

**Figure 2:**
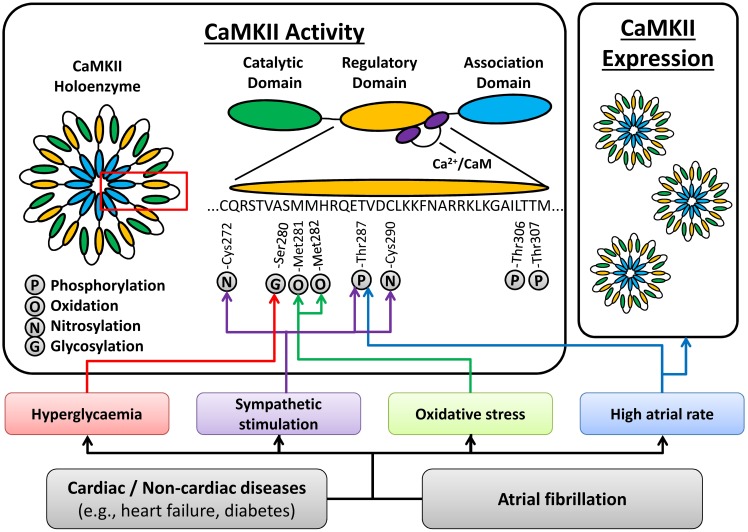
**Atrial fibrillation (AF)-related mechanisms promoting CaMKII activation.** AF-promoting conditions and/or AF itself can activate CaMKII via high atrial rates, oxidative stress, sympathetic hyperactivity, and hyperglycaemia, resulting in post-translational modifications (phosphorylation, oxidation, nitrosylation and glycosylation) of various residues in the regulatory domain of CaMKII. In addition, AF is associated with an increased total expression of the CaMKII holoenzyme. See text for details.

CaMKIIδ protein expression and activity are increased in dogs with pacing-induced atrial tachycardia remodeling (Wakili et al., 2010), goats with long-standing AF (Greiser et al., 2009), and patients with chronic AF (cAF); (Tessier et al., 1999; Neef et al., 2010; Voigt et al., 2012), suggesting that increased CaMKII function can be a consequence of AF. Activation of CaMKII appears to be regulated locally within the myocyte, since autophosphorylation of Thr287 was increased for CaMKIIδ_C_ but not CaMKIIδ_B_ in patients with cAF (Voigt et al., 2012). Several AF-related conditions, including sympathetic hyperactivity, oxidative stress and atrial tachycardia *per se*, may promote CaMKII activation (Figure 2). High atrial-rates during AF can activate CaMKII via frequency-dependent mechanisms. In addition, neuronal autonomic dysbalance can contribute to AF initiation (Park et al., 2012) and atrial tachycardia, in turn, promotes neural remodeling including heterogeneous sympathetic hyperactivity (Jayachandran et al., 2000). Increased sympathetic activity can activate CaMKII through various pathways, including protein kinase-A (PKA)-dependent augmentation of cellular Ca^2+^-cycling (Grimm and Brown, 2010). In addition, PKA-independent, exchange-protein activated by cAMP (Epac) can activate CaMKII following β-adrenoceptor stimulation (Mangmool et al., 2010; Pereira et al., 2013). Moreover, β1-adrenoceptor-activated Epac2 can promote SR Ca^2+^-leak via phosphorylation of RyR2-Ser2814 (Pereira et al., 2013). It has also been suggested that the Epac-mediated CaMKII activation involves phosphorylation of CaMKII-Thr287 by protein kinase-C type-ε (PKCε) (Oestreich et al., 2009) and the upregulation of PKCε in cAF patients (Voigt et al., 2008) might contribute to increased CaMKII activity. Since PKCε translocation to the membrane is increased in atrial myocytes following *in vitro* tachypacing (Makary et al., 2011), this might promote local atrial tachycardia-dependent CaMKII stimulation, although this remains to be proven in future studies. AF is also associated with oxidative stress and oxidation of CaMKII is increased in AF patients (Purohit et al., 2013). Conversely, phosphorylation of the inhibitory Thr306/307 site is decreased in cAF patients, providing another pathway of CaMKII activation in AF (Voigt et al., 2012).

Atrial CaMKII activity is also increased in dogs with ventricular tachypacing-induced heart failure (Yeh et al., 2008), and in goats with atrial dilatation (Greiser et al., 2009), suggesting that CaMKII can be activated by AF-enabling cardiac pathologies, potentially contributing to the evolution of a vulnerable substrate for AF initiation. Similarly, increased body-mass index and diabetes are AF risk-factors (Dublin et al., 2006) that may further promote CaMKII activation via O-linked glycosylation in response to hyperglycaemia (Erickson et al., 2013). Thus, CaMKII activation is multifactorial, resulting from AF itself, as well as from AF-enabling risk factors and diseases (Figure 2).

### Role of CaMKII in ectopic activity

CaMKII has been shown to promote EADs in ventricular cardiomyocytes (Qi et al., 2009), which can produce ectopic (triggered) activity. CaMKII-dependent phosphorylation of I_Ca,L_ slows I_Ca,L_ inactivation, increasing the I_Ca,L_ window current that plays a major role in the generation of EADs (Qi et al., 2009). In addition, the APD-prolonging effects of CaMKII-dependent phosphorylation of I_Na_, increasing I_Na,late_, could further promote the occurrence of EADs and ectopic activity (Wagner et al., 2011). However, since most forms of AF are generally associated with abbreviated APD, the relevance of such EADs may be lower in atrial compared to ventricular arrhythmogenesis. On the other hand, EADs can also arise from Ca^2+^-handling abnormalities that activate depolarizing NCX-current (late phase-3 EADs), which have been implicated in the initiation of AF in some animal models (Burashnikov and Antzelevitch, 2003; Patterson et al., 2006).

Ca^2+^-handling abnormalities can also cause DADs and ectopic (triggered) activity, promoting AF initiation. Genetic mouse models have revealed that intrinsic RyR2-dysfunction is sufficient to increase the susceptibility to pacing-induced AF, as reviewed in (Dobrev et al., 2011). Mice with gain-of-function RyR2 mutations causing catecholaminergic polymorphic ventricular tachycardia (CPVT), and mice lacking the RyR2-stabilizing subunit FKBP12.6, develop Ca^2+^-handling abnormalities including increased SR Ca^2+^-leak and spontaneous SR Ca^2+^-release events (i.e., sparks, waves). These mice also have an increased susceptibility to pacing-induced AF (Sood et al., 2008; Chelu et al., 2009; Shan et al., 2012). Rapid-pacing activates CaMKII and increases CaMKII-dependent RyR2 and PLB phosphorylation. Genetic and pharmacological CaMKII inhibition normalized the susceptibility to pacing-induced AF in mice with a CPVT mutation in RyR2 (Chelu et al., 2009). Of note, selective genetic inhibition of CaMKII-dependent RyR2-hyperphosphorylation (RyR2-Ser2814Ala) also reduced the incidence of rapid-pacing-induced AF in mice where a vulnerable substrate was created using stimulation with the muscarinic-receptor agonist carbachol, and pacing-induced AF in mice deficient of FKBP12.6 (Chelu et al., 2009; Li et al., 2012), strongly suggesting that CaMKII-dependent RyR2 hyperphosphorylation and associated Ca^2+^-handling abnormalities are critical AF-promoting factors (Dobrev et al., 2011). In addition, recent work has identified calmodulin as a direct regulator of RyR2 that stabilizes SR Ca^2+^-release (Yang et al., 2014). Although overall calmodulin levels are increased in cAF patients (Voigt et al., 2012), a reduced affinity between RyR2 and calmodulin, as observed in heart failure (Yang et al., 2014), could potentially contribute to RyR2 dysfunction in AF.

**Figure 3:**
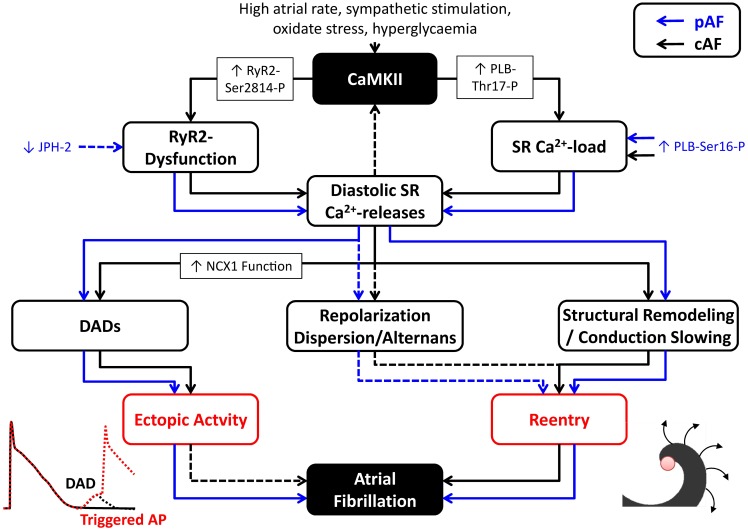
**Proarrhythmic consequences of Ca^2+^/CaMKII dysregulation in atrial fibrillation (AF).** CaMKII activation and CaMKII-dependent phosphorylation of type-2 ryanodine-receptor (RyR2) channels (RyR2-Ser2814-P) and phospholamban (PLB-Thr17-P), as well as other factors, promote spontaneous diastolic sarcoplasmic reticulum (SR) Ca^2+^-release events through RyR2 dysfunction and modulation of SR Ca^2+^-load in patients with paroxysmal AF (pAF; blue lines) and long-standing persistent, chronic AF (cAF; black lines). SR Ca^2+^-leak and diastolic SR Ca^2+^-release events can produce delayed afterdepolarizations (DADs) that contribute to ectopic activity. In addition, they can promote reentry through local repolarization abnormalities, as well as structural remodeling and conduction velocity (CV) slowing. Influences for which the proarrhythmic roles are more speculative have been indicated with dashed lines.

Atrial cardiomyocytes from cAF patients have unaltered RyR2 protein expression levels and SR Ca^2+^-load (Voigt et al., 2012). However, they exhibit CaMKII-dependent RyR2-hyperphosphorylation that increases RyR2 open probability and augments SR Ca^2+^-leak and spontaneous diastolic Ca^2+^-release events. The enhanced SR Ca^2+^ leak results in enhanced DADs and cellular triggered activity and can be blocked using CaMKII inhibitors, thus supporting an important proarrhythmic role for these CaMKII-dependent Ca^2+^-handling abnormalities in human AF (Voigt et al., 2012). In addition, cAF patients had significantly reduced levels of RyR2-stabilizing FKBP12.6 subunits (Vest et al., 2005) and larger transient-inward currents/depolarizations for a given SR Ca^2+^-release. The latter is in part mediated by increased NCX1 mRNA (Gaborit et al., 2005) and protein expression levels (Schotten et al., 2002; El-Armouche et al., 2006; Voigt et al., 2012) in cAF patients. There is evidence that CaMKII can upregulate NCX1 transcription following β-adrenoceptor stimulation (Mani et al., 2010), suggesting that CaMKII could also be involved in the increased NCX1 expression in AF. Although atrial cardiomyocytes from paroxysmal AF (pAF) patients also have increased SR Ca^2+^-leak, spontaneous SR Ca^2+^-release events and DADs, these effects appear to be CaMKII-independent, since CaMKII expression and Thr287 autophosphorylation were not changed in pAF patients (Voigt et al., 2014). Similarly, CaMKII-dependent PLB and RyR2 phosphorylation, as well as NCX1 expression were also unaltered in pAF patients. However, RyR2 expression and RyR2 single-channel open-probability were increased and SR Ca^2+^-load was larger in pAF, likely due to PKA-dependent PLB hyperphosphorylation (Voigt et al., 2014). Computational modeling showed that both increased SR Ca^2+^-load and RyR2 dysregulation contribute to the spontaneous diastolic SR Ca^2+^-release events in cardiomyocytes from pAF patients. Thus, although SR Ca^2+^-handling abnormalities appear a central element in experimental and human AF, the underlying molecular mechanisms are complex. In addition, it is likely that the proarrhythmic consequences of Ca^2+^-handling abnormalities are distinct for different types of AF (Figure 3). Whereas Ca^2+^-mediated triggered activity is a likely candidate for the re-initiation of AF episodes in pAF patients, its relevance for patients with long-standing persistent AF is incompletely understood. In persistent AF forms, Ca^2+^-dependent evolution and progression of atrial remodeling may play a prominent role in arrhythmia maintenance and stabilization (as discussed below).

### Role of CaMKII in reentry-promoting remodeling

Ca^2+^-handling abnormalities also play a role in AF-promoting reentry. APD-shortening is a hallmark feature of AF-related remodeling that facilitates the maintenance of reentrant circuits. It is largely mediated by a reduction in depolarizing I_Ca,L_ and an increase in several repolarizing K^+^-currents. Various mechanisms contribute to reduced I_Ca,L_ in AF (Dobrev et al., 2012). Cav1.2 expression is reduced in AF through a pathway involving the Ca^2+^-dependent phosphatase calcineurin and nuclear factor of activated T-cells (NFAT) (Qi et al., 2008) and increased activation of the Ca^2+^-dependent protease calpain promotes breakdown of I_Ca,L_ channels (Brundel et al., 2004). I_Ca,L_ phosphorylation is also reduced in AF, decreasing current amplitude, and could be due to either increased protein phosphatase activity or local reduction in CaMKII availability (Christ et al., 2004). I_K1_ is increased in cAF patients, and, together with an increase in the acetylcholine-independent “constitutive” activity of I_K,ACh_, results in an overall increase in inward-rectifier K^+^-current that contributes to APD shortening (Dobrev et al., 2005). A Ca^2+^-dependent NFAT-mediated reduction in the inhibitory microRNA-26 in AF results in disinhibition of Kir2.1 expression, contributing to the increase in I_K1_ in cAF patients (Luo et al., 2013). Increased constitutive I_K,ACh_ may also result from Ca^2+^-dependent calpain-mediated reduction in inhibitory PKCα (Makary et al., 2011). Thus, the proarrhythmic increases in I_K1_ and constitutive I_K,ACh_ are partially mediated by Ca^2+^-dependent processes, althouth the potential involvement of CaMKII needs to be specifically addressed in future studies. Finally, the Ca^2+^-dependent small-conductance (SK) K^+^-current (I_SK_) is upregulated in atria of cAF patients, which might contribute to APD shortening (Zhou et al., 2012), although others have reported reduced I_SK_ in AF (Yu et al., 2012). Acute Ca^2+^-dependent regulation of currents such as I_Na_, I_SK_ or I_Ca,L_ can also contribute to beat-by-beat alterations in APD, including APD alternans and augmentation of dispersion of repolarization. These spatial and temporal repolarization heterogeneities favor unidirectional conduction block that can initiate reentry. In agreement, atrial APD alternans is emerging as a clinical index to assess the vulnerability to develop AF in patients (Lalani et al., 2013).

Ca^2+^-entry into atrial fibroblasts via multiple ion channels contributes to fibroblast proliferation and differentiation into collagen-secreting myofibroblasts, which promote fibrosis-induced heterogeneous conduction slowing and reentry (Yue et al., 2011). Transient-receptor potential (TRP) melastatin-related-7 (TRPM7) and canonical-3 (TRPC3) channels are major sources of Ca^2+^-entry into human atrial fibroblasts (Du et al., 2010; Harada et al., 2012). Atrial fibroblasts from AF-patients have larger TRPM7 currents and increased TRPC3 expression, and are more prone to differentiate into myofibroblasts. Knockdown of TRPM7 expression reduces basal differentiation of fibroblasts from cAF patients (Du et al., 2010). Furthermore, pharmacological inhibition of TRPC3 channels reduces AF substrate development and AF duration in dogs with electrically maintained AF (Harada et al., 2012). TRPM7-like channels are inhibited by CaMKII in hepatocytes, which may support hepatocellular survival during proliferation (Mishra et al., 2009). Moreover, Ca^2+^-influx through TRPC3 promotes CaMKII activation and NADPH-oxidase-mediated production of reactive oxygen species in a genetic mouse model (Kitajima et al., 2011). Thus, CaMKII could potentially act both upstream and downstream of TRP channels to alter fibroblast function in AF, although this requires confirmation in subsequent studies.

Ca^2+^-handling abnormalities can also promote reentry by reducing atrial conduction velocity through a reduction in I_Na_ or direct inhibition of gap-junction channels in atrial cardiomyocytes (Heijman et al., 2013b; King et al., 2013b). The reduction in conduction velocity observed in mice with RyR2 mutations could be reproduced in wild-type mice with acute application of caffeine to increase SR Ca^2+^-leak, and appears to be due to both acute Ca^2+^-dependent inhibition of I_Na_, as well as downregulation of Nav1.5 subunit expression under chronic conditions (King et al., 2013a). This Ca^2+^-dependent reduction in I_Na_ is expected to promote reentry-mediated AF maintenance but may also reduce the likelihood of ectopic activity (Heijman et al., 2013b). At present the role of CaMKII in these reentry-promoting Ca^2+^-handling abnormalities is largely unknown, although it has been suggested that CaMKII-dependent phosphorylation could also reduce peak I_Na_, particularly at fast heart rates relevant for AF (Wagner et al., 2006), which could contribute to reentry by reducing atrial conduction velocity.

Cardiac myosin-binding protein-C (cMyBPC) is a critical regulator of myofilament function (Schlossarek et al., 2011). Ser282-phosphorylation of cMyBPC is decreased in dogs with pacing-induced atrial tachycardia remodeling (Wakili et al., 2010), in dogs with ventricular tachypacing-induced heart failure (Yeh et al., 2008), goats with long-standing AF or atrial dilatation (Greiser et al., 2009), and in cAF patients (Tessier et al., 1999; Neef et al., 2010; Voigt et al., 2012). Although there is indirect evidence that this could be due to increased local dephosphorylation by phosphatases, reduced local CaMKII-dependent phosphorylation of Ser282 could also be involved. In addition, contractile dysfunction is promoted by activation of Ca^2+^-dependent proteases. Together, contractile dysfunction and associated atrial dilatation result in a larger vulnerable substrate, promoting reentrant arrhythmias (De Jong et al., 2011).

Accumulating evidence suggests that CaMKII-dependent RyR2-hyperphosphorylation and the related SR Ca^2+^-leak play an important role in AF-promoting structural remodeling. Mice with transgenic overexpression of the transcriptional repressor CREM-IbΔC-X in cardiomyocytes (CREM mice) develop age-dependent progression from spontaneous atrial ectopy to paroxysmal and long-lasting AF episodes (Li et al., 2014). The development of spontaneous AF episodes is preceded by Ca^2+^-handling abnormalities and atrial enlargement. Genetic inhibition of CaMKII-dependent RyR2 phosphorylation (RyR2-Ser2814Ala) in CREM mice prevents Ca^2+^-handling abnormalities and spontaneous AF, as well as atrial dilatation and conduction abnormalities (Li et al., 2014). Thus, CaMKII-dependent RyR2-dysregulation not only contributes to ectopic (triggered) activity, but also drives a progressive development of an AF substrate (Figure 3), promoting atrial hypertrophy and dilatation, and AF progression (Li et al., 2014). These studies suggest the interesting possibility that the progression of AF might be inhibited by targeted treatment of CaMKII or SR Ca^2+^-leak via RyR2. Future studies in mice and large animal models are required to confirm this concept, since the pathophysiological mechanisms and the importance of CaMKII likely vary for different species and experimental AF models, as well as for different forms of clinical AF.

## CaMKII dysregulation and Ca^2+^-handling abnormalities as therapeutic targets in AF

The central role of Ca^2+^-handling abnormalities in AF-pathophysiology suggests their potential as antiarrhythmic targets. Stabilization of RyR2 has emerged as a viable approach to normalize Ca^2+^-handling abnormalities. Several currently-available antiarrhythmic drugs, including the class-Ic Na^+^-channel blocker flecainide (Hilliard et al., 2010), the β-adrenoceptor blocker carvedilol (Zhou et al., 2011), and the antianginal drug ranolazine (Parikh et al., 2012), directly bind and inhibit RyR2 channels. Indeed, flecainide has been successfully employed in other Ca^2+^-dependent arrhythmias such as CPVT (Van Der Werf et al., 2011). However, flecainide also inhibits atrial K^+^-currents like I_K,ACh_ (Voigt et al., 2010), which might contribute to its anti-AF efficacy. More specific RyR2 inhibitors are currently being evaluated in clinical studies (Dobrev et al., 2012).

Inhibition of CaMKII or elimination of CaMKII-dependent RyR2-phosphorylation has proven antiarrhythmic in mouse models of AF and has shown beneficial effects in atrial cardiomyocytes from cAF patients (Chelu et al., 2009; Li et al., 2012; Voigt et al., 2012). However, given the importance of CaMKII in various physiological processes, systemic CaMKII inhibition could have various undesirable side effects, including reduced fertility and impaired memory (Backs et al., 2010; Halt et al., 2012). Moreover, since CaMKII expression/autophosphorylation and CaMKII-dependent phosphorylation of RyR2 and PLB are not increased in pAF patients (Voigt et al., 2014), it is unclear whether CaMKII inhibition would be beneficial for this group of patients. Nonetheless, it appears likely that localized CaMKII inhibition could be a promising antiarrhythmic strategy for appropriately-selected AF patients. Future animal studies and clinical trials will be needed to determine which groups of AF patients are most likely to benefit from CaMKII inhibition. Local inhibition of CaMKII might be possible through inhibition of specific CaMKII-isoforms and splice variants, or by modulating different CaMKII-targeting proteins. Another potential avenue could be the modulation of microRNAs. Injection of complementary “antagomirs” to reduce the activity of certain microRNAs or overexpression of microRNAs has proven beneficial in a variety of experimental models, as reviewed in (Kumarswamy and Thum, 2013). Recent work has shown that CaMKIIδ expression is repressed by microRNA-145 (Cha et al., 2013) and microRNA-30b-5p (He et al., 2013). Increasing the levels of these microRNAs in the heart might, therefore, be an option to inhibit CaMKII.

## Conclusions

Ca^2+^-handling abnormalities promote both focal ectopic (triggered) activity and reentry that contribute to AF initiation and maintenance. The expected increase in the incidence of AF and the limited efficacy and safety of currently available antiarrhythmic drugs, make a better understanding of these AF-modulating processes critical for the development of improved therapeutic strategies. Ca^2+^-handling abnormalities provide a novel set of potential antiarrhythmic targets for the treatment of AF. However, due to the multitude of etiologies and complexity of mechanisms underlying clinical AF, it is likely that tailored therapeutic strategies for specific groups of patients that target multiple pathophysiological processes will be necessary. Cardiac-specific inhibition of CaMKII could be a promising therapeutic strategy for certain groups of AF patients.

## Funding

The authors' work is supported by the European–North American Atrial Fibrillation Research Alliance (07CVD03 to Dobromir Dobrev) and the Alliance for Calmodulin Kinase Signaling in Heart Disease (08CVD01, to Xander H. T. Wehrens) grants of Fondation Leducq, the European Network for Translational Research in Atrial Fibrillation (EUTRAF; 261057, to Dobromir Dobrev), the German Federal Ministry of Education and Research through the DZHK (German Center for Cardiovascular Research, to Dobromir Dobrev), the American Heart Association (13EIA14560061 to Xander H. T. Wehrens), Muscular Dystrophy Association (186530), and National Institutes of Health grants HL089598, HL091947, and HL117641 (to Xander H. T. Wehrens).

### Conflict of interest statement

The authors declare that the research was conducted in the absence of any commercial or financial relationships that could be construed as a potential conflict of interest.
